# Analyzing the Interaction between Anthocyanins and Native or Heat-Treated Whey Proteins Using Infrared Spectroscopy

**DOI:** 10.3390/molecules27051538

**Published:** 2022-02-24

**Authors:** Shuai Ren, Luis Rodriguez-Saona, M. Monica Giusti

**Affiliations:** Department of Food Science and Technology, The Ohio State University, 2015 Fyffe Road, Columbus, OH 43210, USA; ren.313@osu.edu (S.R.); rodriguez-saona.1@osu.edu (L.R.-S.)

**Keywords:** IR, structure change, anthocyanin–whey protein interaction, heat denature, soft independent modeling of class analogy

## Abstract

The color stability of anthocyanins (ACN) has been shown to be improved by interaction with whey proteins (WP). In this study, we explore the ACN–WP interaction using Fourier transform infrared spectroscopy (IR). ACN from purple corn, grape, and black carrot (50 μM) were evaluated. IR spectra (4000–700 cm^−1^) were collected for native and preheated (40–80 °C) WP (5 mg/mL) and ACN–WP mixtures at pH 7.4. Soft independent modeling of class analogy was used to analyze the IR data. The WP secondary structure changed after heat treatments and after interaction with ACN. As expected, the WP α-helices decreased, and β-sheet increased after heat treatment. The intensities of the WP amide I and II bands decreased after ACN addition, revealing a decrease in the WP α-helix content. Higher preheating temperatures (70–80 °C) resulted in a more disordered WP structure that favored stronger WP–ACN interactions related to amide III changes. Addition of ACN stabilized WP structure due to heat denaturation, but different ACN structures had different binding affinities with WP. WP structure had less change after interaction with ACN with simpler structures. These results increase our understanding of ACN–WP interactions, providing a potential strategy to extend anthocyanin color stability by WP addition.

## 1. Introduction

Anthocyanins (ACN) are water-soluble pigments widely used as food colorants in the food industry to produce colors from red to purple to blue [1]. ACN are found in flowers, fruits, vegetables, and tubers. The basic structure of ACN consists of 2-phenyl-benzopirylium or flavylium with a number of hydroxy and methoxy [2]. A wide range of biological activities has been reported for ACN, such as antioxidant, anti-inflammatory, antiviral, antimicrobial, antimutagenic, and antitumor activities, and ACN are also associated with inhibition of some disorders such as cancer, cardiovascular diseases, diabetes, and obesity [3,4].

Utilization of ACN as food colorants has been limited by their low stability and interaction with other compounds in the food matrix [5]. Various factors affect ACN color and stability, such as the temperature, pH, light, oxygen, enzymes, metallic ions, flavonoids and phenolics, ascorbic acid, sugars, sulfites, chemical structure, and concentration of the ACN [6,7]. It is valuable and challenging to find an effective way to reduce ACN losses during food processing and storage.

The complexation between whey proteins (WP) and ACN has received increasing attention, and it is believed to be explained mainly by noncovalent binding [8,9]. The major fraction of WP that interacts with ACN is the globular protein β-lactoglobulin (β-LG). β-LG exists as a dimer at neutral pH and folds into a calyx form by antiparallel β-sheets [10]. It possesses a hydrophobic pocket located in the interior of the calyx structure that serves as the primary binding site for the ligands. Thus, β-LG displays a strong binding affinity for various hydrophobic and amphiphilic ligands, including fatty acids, retinol, β-carotene, phospholipids, vitamin D, folic acid, and phenolic compounds [11]. Heat treatment denatures proteins and promotes their conformational modifications at the secondary and tertiary structure levels, which affects their hydrophobicity, functional properties, and the ability to bind other molecules [12]. More disordered proteins have stronger interactions with phenolic compounds than globular proteins [13]. Thus, the thermally modified WP is expected to have higher binding efficiency with ACN. In a previous study, we showed that preheated WP had higher binding affinity with ACN than native WP and the binding affinity decreased with increasing WP preheating temperatures [14]. Another previous study showed that WP addition improved ACN color stability during storage, with the smallest color change occurring when WP was preheated to 50 °C [15]. A previous study also showed that preheated WP effectively increased its protective effects on the thermal, oxidation, and photostability of grape skin ACN extracts and the optimal effects occurred when WP were preheated at 50 °C for 15 min [16]. Different ACN sources might have different binding affinities and binding forces with WP. Based on our previous studies, purple corn ACN were mainly monoglycosylated cyanidin derivatives, with ~14% of those pigments being acylated with malonic acid. Grape ACN mainly contained malvidin-diglucosylated derivatives, with ~16% ACN acylated with p-coumaric acid. Black carrot were rich in tryglycosylated cyanidin-derivatives, with ~80% ACN acylated with cinnamic acids. The purple corn ACN had higher binding affinity than grape and black carrot ACN due to their simpler ACN structure [14]. Purple potato ACN interacted with WP mainly through Van der Waals force and hydrogen bond [17], while blueberry ACN bond with WP mainly by hydrophobic forces [18].

Fourier transform infrared spectroscopy (IR) provides information about the secondary structure of proteins and is widely used in protein structure and conformation analysis because of its ease of operation, rapid output, high light intensity at the detector, high signal-to-noise ratio, and non-destructive sample preparation [19]. Proteins have two major absorption bands in the mid-infrared (MIR) region, the amide I and amide II bands [20]. The amide I band primarily arises from the C=O stretching and the absorbance is found at approximately 1650 cm^−1^. The amide II band is mainly due to a coupled N–H bending vibration and C–N stretching vibration, which absorbs at approximately 1550 cm^−1^ [21]. The locations of both the amide I and amide II bands are sensitive to the secondary structure content of a protein because the hydrogen bonding takes place on both the C=O and the N–H bonds between the different elements of secondary structure [22]. A previous IR study showed that after the binding between both native and preheated β-LG with cyanindin-3-glucose, β-LG structure had a reduction in the proportion of α-helix and β-sheet structures accompanied by an increase in the proportion of random coil and turn structures [8].

The binding affinity between WP and ACN is expected to change according to different WP secondary structures and ACN sources. IR can provide information about whether the secondary structure of WP is also changed after binding with ACN. Therefore, the objective of this study was to explore the effect of the heat-induced WP structural changes and ACN structure on the ACN–WP interaction by IR. Three ACN sources (purple corn, grape, black carrot) with different ACN compositions and commercial values were chosen for examination in this study. The hypothesis is that the WP structure will become more unfolded with increasing preheating temperature, which results in different binding affinity with ACN. ACN with more complex structures will have weaker binding with WP due to the difficulty to enter the WP hydrophobic pocket. These results may contribute to a better understanding of how to use WP to enhance the stability of ACN.

## 2. Results and Discussion

### 2.1. The Effect of Heating on Whey Protein Structure

**Figure 1:**
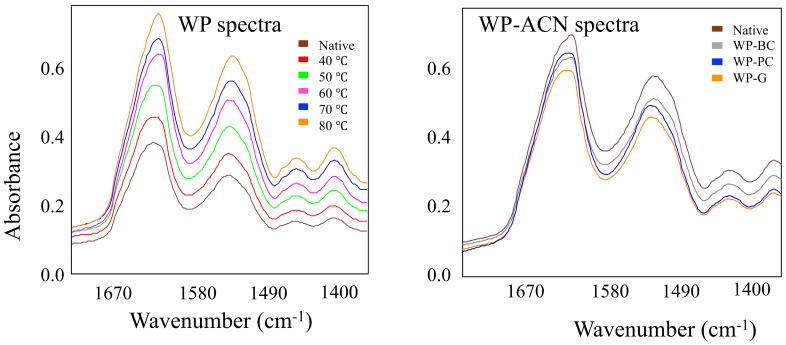
Infrared spectra of native whey protein (WP) and WP subjected to different heat treatments with no anthocyanins added (**left**) and spectra of native WP before and after addition of anthocyanin (ACN) extracts (**right**). Spectra collected on a 4500 series mid-IR spectrometer. BC is back carrot anthocyanin, PC is purple corn anthocyanin, G is grape anthocyanin.

The IR spectra of native WP, as well as the spectra of WP samples heated at different temperatures are shown in Figure 1. WP showed two distinct bands associated with functional group vibrations in the information-rich region of 1700–1400 cm^−1^. The major band in the raw spectrum was centered at approximately 1640 cm–1 and was associated with the amide I. This band is related to *α*-helix and *β*-sheet structures of proteins [23]. The second major band was centered at about 1540 cm^−1^ and corresponded to the amide II, which is mainly attributed to NH in plane bend and CN stretching vibrations [21]. Additional bands were observed at around 1450 and 1400 cm^−1^ resulting from the CH in plane bending vibration in the CH_2_ groups and the C=O stretching vibration of COO^−^ groups [21]. There were no evident differences in the spectral patterns because of the preheat treatment of the WP or by addition of anthocyanins, but the peak intensity increased with increasing WP preheating temperatures (Figure 1). In order to elucidate the changes occurring by the treatments, the data needed to be analyzed by pattern recognition analysis to extract relevant information from the spectra.

**Figure 2:**
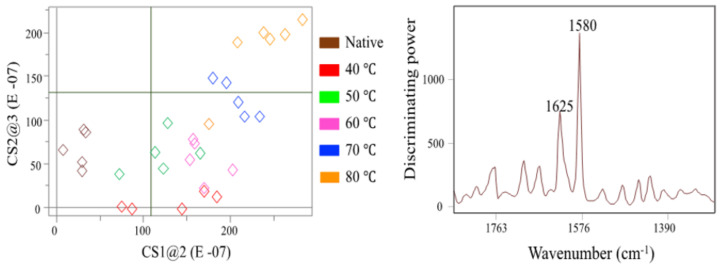
Soft independent modeling of class analogy (SIMCA) Coomans’ plots (**left**) and discriminating power (**right**) based on the IR spectra of native WP and WP heated to different temperatures (no anthocyanin added).

Figure 2 shows the SIMCA Coomans’ plot that assessed the classification performance of the classification model by determining class membership in terms of distance from the boundaries (95% confidence limits) of the categories generated by the supervised model. In a Coomans’ plot, the two axes represent the distance of each sample from a specific class (e.g., native WP or heat-treated WP at 40 °C), so that each class model is drawn as a rectangle corresponding to the critical distance (*p* = 0.05) from the class. Any sample having a distance to the corresponding class rectangle greater than the critical distance is considered as being outside the class model and, therefore, rejected as an outlier from the specific class (graphically, it is plotted on the top right quadrant). Moreover, the samples plotted onto the lower left quadrant of the diagram are assigned to both classes. Figure 2 indicates the similarities between the native WP and samples heat-treated for 40 °C and 50 °C. This finding is supported by the low interclass distances (Table 1) of ~0.5 for classes 1 (Native), 2 (WP heat-treated for 40 °C) and 3 (WP heat-treated for 50 °C). Interclass distance, a statistically derived measure (Euclidean distance) of how far away clusters are from each other [24,25], greater than 3 is considered significant for identification of data points as members of different groups [26]. WP samples heated at temperatures above 60 °C resulted in distinctive spectral changes among groups of samples (interclass distances > 2.3). This result matched with the fact that regular structures of protein will convert into more unfolded and random structure with increasing heating temperature [27]. By employing the discrimination power statistic (Figure 2), we identified the bands centered at 1625 cm^−1^ (amide I) and 1580 cm^−1^ (amide II) as responsible for the changes in WP due to the heating treatments. The absorbance intensity of the amide I band is predominantly due to the C=O stretching vibration with minor contributions from the out-of-phase C–N stretching vibration, the C–C–N deformation and the N–H in-plane bend [21]. The amide I band centered at 1633 cm^−1^ is characteristic of proteins rich in *β*-sheet structures [28]. The amide II band centered at 1580 cm^−1^ is related to the out-of-phase combination of the N–H in-plane bend and the C–N stretching vibration with smaller contributions from the C–O in plane bend and the C–C and N–C stretching vibrations [21]. Thus, when proteins are subjected to increasing temperatures, the more regular structures (*α*-helix, *β*-sheet) of WP are converted into irregular (unfolded) structures [27]. 

### 2.2. The Effect of Anthocyanin Addition on Whey Protein Structure

**Figure 3:**
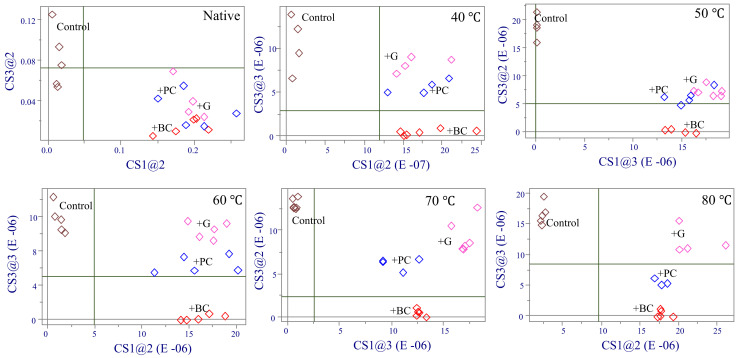
Coomans’ plots comparing whey protein spectra before and after addition of and three anthocyanin (ACN) mixtures at different preheating temperatures. Control sample (no ACN, brown), black carrot ACN (red), purple corn ACN (blue), grape ACN (pink).

Figure 3 shows the Coomans’ plot for preheated WP after incorporation of three ACN (purple corn, black carrot, and grape) extracts. All Coomans’ plots contrasted the same treatments, control (class 1, no ACN addition) vs addition of purple carrot extract (class 2), for the different preheating temperatures to which the WP was exposed. Although addition of the anthocyanin extract to the native WP solution resulted in spectral changes, all shared the same chemical characteristics. However, the type of anthocyanin added to the pre-heated WP showed an important effect on the way anthocyanin pigments interacted with the denatured WP (Figure 3). For each WP preheated at different temperatures, there were four well separated clusters among samples, indicating that different ACN structures had different binding affinity with WP and produced different ACN–WP complexes.

**Figure 4:**
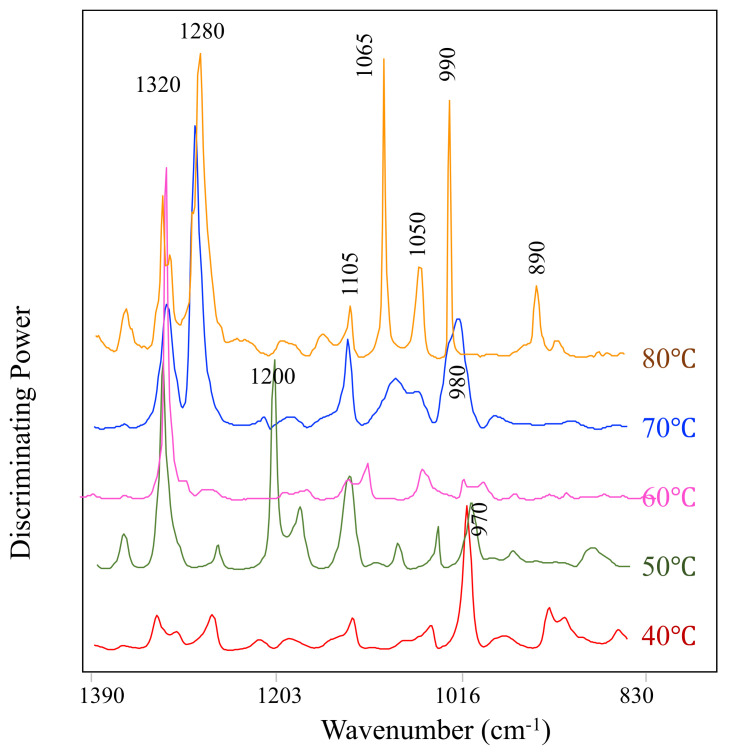
Discriminating power for comparison between WP alone vs WP–ACN mixtures at different preheating temperatures.

To elucidate the chemical changes accounting for the clustering of the treatments, the discriminating power was used to identify the most relevant variables responsible for the classification (Figure 4). Discrimination power shows the effect of ACN addition to the WP solution regardless of the type of ACN. At low temperature (40 °C), the main differences were explained by the band at 970 cm^−1^, typical of the skeletal vibrations in glycosidic and pyranose rings of sugars [29]. Qin and others (2018) reported that glucose molecules could graft on the surfaces of proteins and might form a barrier that prevents polyphenol–protein interactions [30]. Increasing the preheating temperature to 50 °C showed a more complex profile of bands having a discriminating effect, with major bands at 970 and 1105 cm^−1^ associated with sugar moieties, 1200 cm^−1^ assigned to the –OH bond of flavonoids, and 1320 cm^−1^ related to the amide III mode corresponding to α-helix structures. Interestingly, the discriminating power for anthocyanins mixed with WP pre-heated at 60 °C only showed the band at 1320 cm^−1^ as important for cluster separation. Pre-heating WP solutions at temperatures of 70 °C and 80 °C before adding the anthocyanin extracts showed a shift of the bands centered at 970 cm^−1^ to 980–990 cm^−1^ related to the methylation (OCH_3_) vibrations of sugars, band absorptions at 1050, 1065, and 1105 cm^−1^ due to the C–OH stretching vibration of sugars [31]. This data indicate that the sugars attached to the ACN may be playing a role in the ACN–WP interaction, likely through hydrogen bonds. In addition, the bands at 1280 and 1320 cm^−1^ had a major effect at temperatures > 70 °C and were attributed to the heat-induced changes in the amide III region of the protein structure [32]. We also observed a shift on the sugar bands. Zang and others (2021) showed that different numbers of the hydroxyl groups in ACN interacted with the C=O and C–N groups of WP, resulting in a rearrangement of the peptide chain of WP and hence a change in WP secondary structure [18]. Thus, the different ACN structures can have different binding affinity with WP.

### 2.3. The Effect of Anthocyanin Structure on Anthocyanin–Whey Protein Interaction

**Figure 5:**
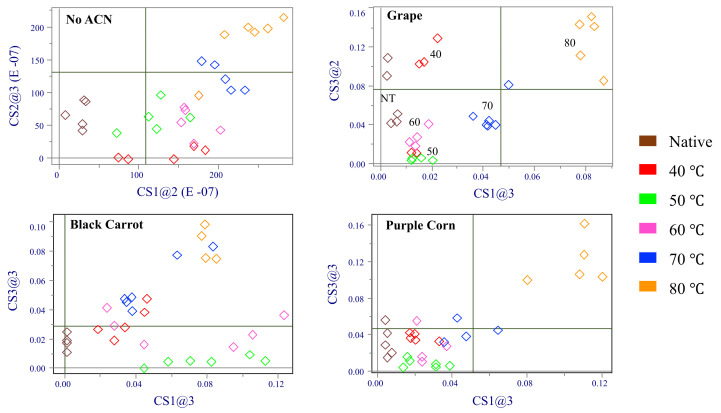
Coomans’ plots comparing the interaction of different anthocyanins (ACN) with native or preheated WP. Black carrot ACN (red), purple corn ACN (blue), grape ACN (pink).

Coomans’ plots comparing the interaction of different anthocyanins with native or preheated WP are shown in Figure 5. When no ACN was added, WP samples were separated into three different quadrats due to their structure change. After ACN was added, WP preheated at 40, 50, 60, and 70 °C tended to stay in the same quadrat with native WP, especially for purple corn ACN. This result identified that addition of ACN could stabilize WP’s structure. Although the WP heated at 80 °C showed significant differences with samples in other preheat temperatures, the scale in Coomans’ plot decreased from 50 to 0.04 after addition of ACN proving that the WP structure had fewer changes at the same preheating temperature after binding with ACN. For purple corn ACN–WP samples, all preheated samples were in the same quadrant with the control, except for WP heated at 80°C. Compared with other two ACN, purple corn ACN showed the least effect on WP structure change, which was due to its simpler ACN structure [33]. WP preheated at higher temperatures had greater effects for WP–ACN structure change. As shown in Figure 5, there was an increasing distance for preheated WP samples to the native control with increasing the WP preheating temperatures from 40 to 80 °C.

**Figure 6:**
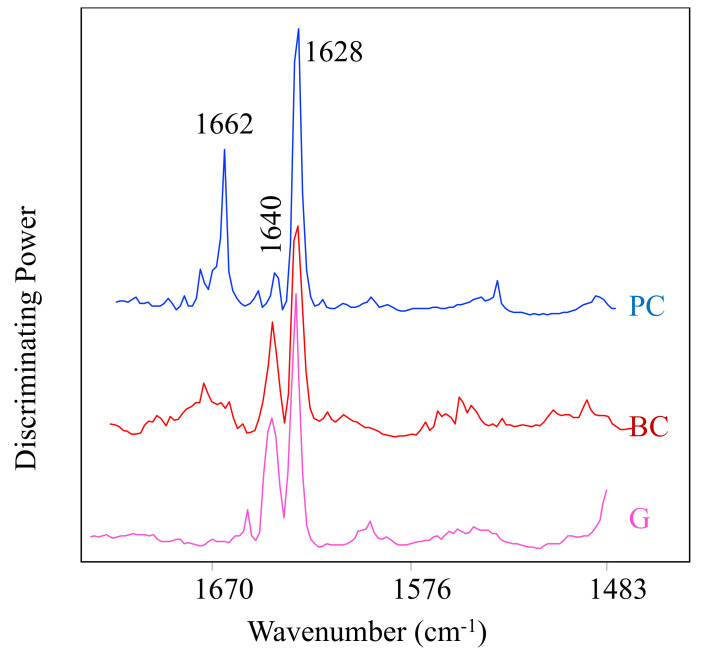
Discriminating powers for different anthocyanins (ACN) with preheated WP, regardless of the preheating temperature. BC: black carrot ACN (red), PC: purple corn ACN (blue), G: grape ACN (pink).

Figure 6 showed the discriminating power for different ACN with preheated WP, regardless of the preheating temperature. For all three ACN, the different samples were mainly discriminated by two bands at 1628 and 1640 cm^−1^, which might correspond to the C=C aromatic and C=O group changes in the protein amide I structure [21]. Based on previous study, in *β*-LG, the bands at 1625 cm^−1^ could be associated with protein aggregation, which is the formation of intermolecular hydrogen bond in *β*-sheet (low frequency) during heat denaturation. The bands at 1650–1660 cm^−1^ due to *α*-helices decrease was observed in both heated and high-pressure treated samples [34]. Therefore, for grape and black carrot ACN, the band at 1640 and 1628 cm^−1^ suggested *β*-sheet structures change in protein aggregation. For the purple corn ACN, another band at 1662 cm^−1^ might be related to *α*-helices decrease in WP during heat treatment. Those results suggested that for each ACN, different preheating temperature changed the secondary structure of WP and therefore affected ACN–WP interaction.

## 3. Materials and Methods

### 3.1. Materials and Chemicals

Three ACN-rich sources with different anthocyanin profiles were evaluated in this study. Purple corn ACN powder, rich in cyanidin-3-glucoside with and without malonic acid acylation, was obtained from Artemis International, Inc. (Fort Wayne, IN, USA). Grape juice ACN concentrate, rich in malvidin-3,5-diglucoside with or without coumaric acid, and black carrot ACN juice concentrates, rich in cyanidin-3-tryglycosides acylated with hydroxycinnamic acids, were obtained from DDW, The Color House (Port Washington, WI, USA). Grass-Fed WP isolate (less than 1% non-GMO sunflower lecithin) was purchased from ProMix Nutrition (Gainesville, FL, USA). The purity of WP was about 91%. The chemicals and reagents (ACS or HPLC grade) were purchased from Fisher Scientific (Fair Lawn, NJ, USA), including methanol, hydrochloric acid (HCl), sodium phosphate dibasic (Na_2_HPO_4_), potassium dihydrogen phosphate (KH_2_PO_4_), sodium chloride (NaCl), potassium chloride (KCl), and sodium acetate. ACS grade ethyl acetate was obtained from Mallinckrodt Chemicals (Bedminster Township, NJ, USA). Anhydrous ethanol 200 PRO was purchased from Decon Laboratories, Inc (King of Prussia, PA, USA).

### 3.2. Buffer System and Sample Preparation

Although ACN was more stable in acidic pH, the COO^−^ groups in acid molecules will interfere the WP IR signal. Therefore, phosphate buffered saline (PBS) (1X, pH 7.4) was used as the buffer system in this study, because no signal from the buffer could be detected by IR. The buffer solution was prepared by mixing 0.137 M NaCl, 0.0027 M KCl, 0.01 M Na_2_HPO_4_, and 0.0018 M KH_2_PO_4_ according to AAT Bioquest Inc. (2020) [35]. Commercial WP isolate powder was dissolved in PBS buffer to reach a concentration of 5 mg/mL. The WP solutions were preheated in a water bath (Fisher Scientific, Fair Lawn, NJ, USA) at 40 °C, 50 °C, 60 °C, 70 °C, or 80 °C for 30 min and then quickly cooled in ice. The preheated WP solutions and their unheated counterpart (native WP) were stored at 4 °C until analysis.

Purple corn, grape, and black carrot ACN were prepared as described in our previous study [14]. They were dissolved in acidified (0.01% *v*/*v* HCl) distilled water and semi-purified by solid phase extraction C-18 cartridge. Semi-purified pigments were redissolved in acidified distilled water and stored at –18 °C to minimize pigment degradation. The pH differential method was used to determine monomeric ACN concentration [36]. Specifically, ACN sample was diluted with 0.025 M potassium chloride buffer pH 1, until the absorbance of the sample at the λ_max_ was within the linear range of the spectrophotometer. The dilution factor was calculated by dividing the final volume of the sample by the initial volume. Then two dilutions of the ACN sample, one with pH 1 potassium chloride buffer and the other with 0.4 M pH 4.5 sodium acetate buffer, were prepared according to the previously determined dilution factor. After 15 min equilibrium, absorbance of each dilution was measured at 700 nm and its λ_max_. Measurements were performed in triplicates, and each ACN pigment was expressed as cyanindin-3-glucoside equivalence. The absorbance of the diluted sample (A) was calculated by

A = (A _(λvis-max)_−A_700_) _pH 1_−(A _(λvis-max)_−A_700_)_pH 4.5_ (1)

and the monomeric ACN pigment concentration in the sample was calculated by

Monomeric ACN pigment (mg/liter) = (*A* × *MW* × *DF* × 1000)/(ε × 1) (2)

where *MW* is the molecular weight, *DF* is the dilution factor, and ε is the molar absorptivity (for cyanindin-3-glucoside, *MW* = 449.2 and ε = 26,900).

Each semi-purified ACN extract was mixed with the preheated or native WP solutions at room temperature. The final concentration of WP in the mixtures was 5 mg/mL and the ACN concentration was 50 μM. The addition of ACN did not appreciably modify the pH of the protein solutions. The mixtures were equilibrated for 15 min before analysis. The IR spectra were collected for native WP, preheated WP, ACN extracts, and all ACN–WP mixtures at room temperature. Experiments were conducted at a pH of 7.4, where ACN may degrade quickly, therefore ACN–WP mixtures were prepared fresh right before analysis.

### 3.3. Fourier Transform Infrared Spectra Measurement

Infrared spectra analysis was performed using an Agilent 4500 portable IR spectrometer (Santa Clara, CA, USA) equipped with a Michelson interferometer, zinc selenide beam splitter, low-powered solid-state laser, wire-wound element infrared source, and thermoelectrically cooled deuterated-triglycine sulfate (DTGS) detector. The mid-infrared 4500 IR spectrometer was interfaced with an attenuated total reflectance (ATR) accessory equipped with a triple reflection diamond crystal with a 2 mm diameter sampling surface and 200 μm active area providing 6 μm effective penetration depth for IR energy at 1700 cm^−1^. Spectra of extracts, WP, and ACN–WP samples were recorded using MicroLab software (Agilent Technologies, Santa Clara, CA, USA) in the spectral range of 4000–700 cm^−1^ at a resolution of 4 cm^−1^ with a total of 64 scans. The diamond crystal was carefully cleaned with 70% ethanol before each measurement to avoid carryover of biomass from previous samples. The spectrum of the empty crystal was used as background. The protein contents were analyzed based on amide I band (~1650 cm^−1^) and amide II band (~1550 cm^−1^).

### 3.4. Statistical Analysis

All the analyses were performed in triplicates. The IR spectra collected were analyzed by soft independent modeling of class analogy (SIMCA), using the chemometrics modeling software Pirouette (v4.5, Infometrix Inc., Woodville, WA, USA).

## 4. Conclusions

IR spectroscopy provided evidence that the WP secondary structure changed due to different heat treatments, particularly with higher temperatures. In addition, WP secondary structure was affected by addition of ACN, with different degrees of WP affinity for different types of anthocyanins. The discrimination power plots confirmed that the classification among different WP samples was mainly based on the *α*-helices decrease and *β*-sheet increase in WP secondary structure. Increasing heating temperatures increased interclass distances between native WP and thermally treated proteins, and also between WP and ACN–WP mixtures, indicating that the more disordered WP structures at high temperatures (70–80 °C) had higher affinity for ACN. At low temperatures (native WP or 40–50°C preheated WP), sugar in ACN played the main role in discriminating ACN–WP samples. At higher temperatures, the main differences between WP and WP–ACN mixtures were more related to WP Amide III change. The secondary structure of WP changed after ACN addition and the structural change varied for the different ACN extracts. Black carrot ACN showed more difference than the other two ACN because of their more complex triacylated structure. The intensities of the amide I and II bands of WP decreased after binding with ACN, revealing that its *α*-helix content in the protein structure was decreased. The addition of ACN also could stabilize WP structure change due to heat denaturation. Purple corn ACN–WP samples showed the best ability to stabilize WP structure, probably due to their simpler molecule structure with only one sugar. All those results support our hypothesis and can improve our understanding on ACN–WP interaction, thus providing a potential method to extend color stability of ACN with WP addition.

## Figures and Tables

**Table 1:** Interclass distances generated from native and differently preheated WP calibration model using 4500 series Attenuated Total Reflectance (ATR) mid-IR spectrometer.

	Native	40	50	60	70	80
Native	0.000					
40	0.499	0.000				
50	0.547	0.513	0.000			
60	2.307	3.195	2.801	0.000		
70	3.468	3.606	3.591	3.327	0.000	
80	7.720	8.144	8.366	6.866	4.110	0.000

## Data Availability

Not applicable.

## Sample Availability

Not available.
